# Genomics meets proteomics: identifying the culprits in disease

**DOI:** 10.1007/s00439-013-1376-2

**Published:** 2013-10-18

**Authors:** Hendrik G. Stunnenberg, Nina C. Hubner

**Affiliations:** Department of Molecular Biology, Faculty of Science, Nijmegen Centre for Molecular Life Sciences, Radboud University Nijmegen, 6525 GA Nijmegen, The Netherlands

## Abstract

Genome-wide association studies (GWAS) revealed genomic risk loci that potentially have an impact on disease and phenotypic traits. This extensive resource holds great promise in providing novel directions for personalized medicine, including disease risk prediction, prevention and targeted medication. One of the major challenges that researchers face on the path between the initial identification of an association and precision treatment of patients is the comprehension of the biological mechanisms that underlie these associations. Currently, the focus to solve these questions lies on the integrative analysis of system-wide data on global genome variation, gene expression, transcription factor binding, epigenetic profiles and chromatin conformation. The generation of this data mainly relies on next-generation sequencing. However, due to multiple recent developments, mass spectrometry-based proteomics now offers additional, by the GWAS field so far hardly recognized possibilities for the identification of functional genome variants and, in particular, for the identification and characterization of (differentially) bound protein complexes as well as physiological target genes. In this review, we introduce these proteomics advances and suggest how they might be integrated in post-GWAS workflows. We argue that the combination of highly complementary techniques is powerful and can provide an unbiased, detailed picture of GWAS loci and their mechanistic involvement in disease.

## Introduction

A human cell is defined by its components, such as the genome, epigenome, proteome, metabolome or transcriptome, and their interactions. This results in a complex regulatory network that we just begin to understand and that poses a major challenge in finding the cellular cause of a given human disease. Even though a systems biological approach integrating all aspects that define a cell type would be best suited to understand human development and disease, researchers only slowly start to leave the isolation of their own specialized -Omics domain.

The field of genomics is likely the most advanced in its global search for disease-associated alterations of the genome. Already for decades, inheritance studies based on genetic linkage in families have been used to map genomic loci that have an effect on disease or other phenotypic traits. Linkage analysis relies on the co-segregation of marker alleles, which are, for example, common single nucleotide polymorphisms (SNPs) with the unknown disease gene within pedigrees. While this approach has had great success for diseases and traits that are controlled by a single locus (Mendelian traits) (Botstein and Risch 2003), it has proven cumbersome for the analysis of common and complex diseases such as cancer (Altmuller et al. 2001). Already in 1996, Risch and Merikangas proposed the performance of an association scan that involves millions of common variants of the genome and a group of unrelated individuals that differ in a certain phenotype. In particular for complex traits this approach should yield much better results than a linkage analysis including only a few hundred markers (Risch and Merikangas 1996). Based on this principle, the first genome-wide association study (GWAS) published in 2005 (Klein et al. 2005) marks the beginning of a whole new era of research counting 1,600 published GWA reports and 10,088 disease-associated SNPs by May 2013 (Hindorff LA 2013).

Even though bearing great promise, the success of GWAS for clinical benefits such as the discovery of new biomarkers that can be used for clinical decision support or disease prevention remains limited. There are two main reasons for this: First, the problem of missing heritability and second, the limited identification and functional characterization of causal variants. Heritability is defined as the proportion of the phenotypic variance in a population that is due to genotypic differences among individuals (Gibson and Shepherd 2012). For example, human height has an estimated heritability of 80 %, meaning 80 % of height differences between individuals can be explained by genetic differences and 20 % are due to other influences such as the environment. Even though 40 genomic loci have been identified to be associated with human height, they only explain 5 % of the phenotypic variance (Visscher 2008). Multiple reasons have been suggested to explain the missing heritability, one of them being the fact that GWA studies typically identify common variants (present in 5 % or more of the population) with small effects and miss out on rare variants (allele frequency <1 %) with potentially much larger effects. This topic is extensively reviewed in Manolio et al. (2006) and Gibson (2011).

In this review, we will focus on the second aspect: The identification and, in particular, the functional characterization of causal variants. Principles for the post-GWAS functional characterization of risk loci are also reviewed elsewhere (Freedman et al. 2011); however, possibilities that mass spectrometry-based proteomics can offer are not discussed. We will summarize both (epi-)genomics and proteomics technology in light of post-GWAS, and thus hope to provide a basis for an highly integrated, systems biological approach. As we cover multiple broad topics, we apologize that due to space restrictions we were not able to cite all relevant publications and would like to refer to other reviews cited in the text. Throughout the review, we mainly discuss SNPs or small genomic variants, but we recognize that other types of common genetic variation, such as larger insertions or deletions, may also influence risk.

## Identification of all common and rare associated variants

If a certain combination of genomic loci in a population occurs more or less often than would be expected from a random formation, they are defined to be in linkage disequilibrium (LD) with each other (reviewed in Slatkin 2008). It is a second-order phenomenon derived from linkage, which is the presence of two or more loci on a chromosome with limited recombination between them. SNPs represented on a GWAS SNP array were chosen in a way that they capture the LD structure of the genome and thus allow the identification of associations between a common trait and a certain genomic region that is represented by one marker (tagSNP). Hence the associated SNP is not always the causal variant and any other SNP or a combination of SNPs that are in strong LD with the tagSNP can form the basis of functional consequences. For this reason, one of the major tasks of the HapMap project is to identify all common and rare variants to generate a comprehensive catalog of human genome variations (Altshuler et al. 2010).

In 2012, the 1,000 genomes project consortium published genomes of 1,092 individuals from diverse ethnic populations using a combination of low-coverage whole-genome and exome sequencing (Genomes Project Consortium 2012). This study captures up to 98 % of accessible SNPs that have a frequency of 1 % or higher in UK-sampled genomes and with 38 million SNPs and approximately 1.5 million other variants provides an extensive resource of common and rare variants. Trait-associated SNPs that are in LD with a certain tagSNP can thus be imputed (Howie et al. 2012). While this approach shows great success for common variants (>5 % frequency), rare variants are more recent and thus geographically restricted. For this reason, many more individuals from different populations around the globe need to be sequenced to provide good coverage. In addition, large efforts are currently taken to fine map regions that are associated with a certain disease phenotype by extensive targeted re-sequencing (Nejentsev et al. 2009; Rivas et al. 2011).

## Identification and characterization of the functional variants

Having successfully identified thousands of novel common and rare variants that are in LD with previously characterized GWAS tagSNPs, the next big challenge is to find the causal variants amongst those. Most methods that have been developed so far focus on SNPs that are located in the coding or transcribed region of a gene because these might influence the primary structure and thus the function of a protein (Ng and Henikoff 2003; Saccone et al. 2011; Cvejic et al. 2013). However, most of the associated common variants identified so far do not map within or in LD to a protein coding region (Easton et al. 2012) and thus might be rather linked to gene expression regulatory mechanisms. Their characterization remains difficult and requires the integration of data from related fields such as epigenetics or proteomics.

### Integration of GWAS with epigenetics information on regulatory elements

A SNP located in a non-coding region may, for example, disrupts or creates a transcription factor (TF)-binding site in an active regulatory element (Reddy et al. 2012). As a consequence, the regulatory activity and thus the expression of a gene that is controlled by this element can be altered (Kasowski et al. 2010). A GWAS SNP that overlaps with an active regulatory region or an experimentally detected TF-binding site in a relevant cell type therefore has a higher probability of being functionally relevant (Jia et al. 2009; Harismendy et al. 2011; Paul et al. 2011).

In fact, a recent study involving genome-wide DNase I mapping in 349 cell and tissue samples showed that 76.6 % of all non-coding GWAS SNPs either lie within a DNase I hypersensitive site (DHS) or are in complete LD with SNPs in a nearby DHS (Maurano et al. 2012). Besides studying histone modifications and the binding pattern of TFs by chromatin immunoprecipitation followed by sequencing (ChIP-seq) (Johnson et al. 2007; Robertson et al. 2007), DNase I hypersensitive site identification by sequencing (DNase-seq) or digital genomic footprinting (DGF) (Crawford et al. 2006; Boyle et al. 2008; Hesselberth et al. 2009) are major techniques to map regulatory elements (Visel et al. 2009). We recently used the combination of these technologies to define binding sites and thus the regulatory impact of the oncofusion proteins PML-RARα and AML1-ETO in acute myeloid leukemia (Saeed et al. 2012).

Large epigenetics consortia such as ENCODE (http://genome.ucsc.edu/ENCODE/), Roadmap (http://www.roadmapepigenomics.org/), iHEC (http://www.ihec-epigenomes.org/) or BLUEPRINT (http://www.blueprint-epigenome.eu) utilize next-generation sequencing technology to characterize, amongst others, histone modifications, TF binding and chromatin accessibility in various cell types, human tissue and blood, respectively. This enormous resource can be used to characterize the regulatory landscape of susceptibility regions in relevant cell types and narrow down causal variants to those mapping to an active regulatory element. RegulomeDB is a database that combines data from ENCODE and other sources such as ChIP-seq data from the sequence read archive (SAR) (Leinonen et al. 2011) and data on expression quantitative trait loci (eQTL) with computational predictions to estimate the regulatory potential of a certain genomic locus (Boyle et al. 2012). A parallel publication of the same group shows that SNPs that are annotated with a high score are in most occasion SNPs that are in LD with a reported association rather than the tagSNP itself (Schaub et al. 2012b).

### Identification of physiological relevant target genes

As mentioned above, one of the major mechanisms underlying susceptibility to complex trait or disease is probably the variation in gene expression caused by polymorphisms in regulatory elements. Consequently, transcript abundance can be analyzed with genetic approaches in the same way as any other quantitative trait phenotype, such as height or the body mass index and are commonly known as expression quantitative trait loci (eQTLs) (reviewed by Cookson et al. 2009). A SNP in a non-coding genomic region could thus be linked to a certain phenotypic trait in a GWA study and the same SNP or a SNP in strong LD could be linked to expression changes in an independent eQTL study, thus providing an important connection between a phenotypic trait and a physiological relevant target gene.

Mapping eQTL target gene associations in tumors is more challenging than for other human traits or disease. Tumors acquire frequent genetic and epigenetic alterations, which can substantially affect gene expression (Raval et al. 2007; Smith et al. 2006) and consequently obscure the association between germline genetic polymorphisms and gene expression (Curtis et al. 2012). For these reasons, recent cancer studies also investigate the association between SNPs and an altered epigenetic landscape, such as promoter methylation, histone modifications or the expression of large intergenic non-coding RNAs (lincRNAs) that associate with chromatin-modifying complexes (Gibbs et al. 2010; Bell et al. 2011; Grossman et al. 2013; Ernst et al. 2011). A good example is the recent work from Li et al. (2013), which provides a more comprehensive picture of gene expression determinants in breast cancer and the underlying biology of breast cancer risk loci by the integrated analysis of eQTLs, somatic copy number alteration and CpG methylation in 219 tumor samples and the healthy counterparts.

Even though quantitative trait loci analysis can indicate an impact of a genomic region on the expression or the epigenetic regulation of certain genes, it is unclear if this influence is direct or indirect. Mammalian genomes are organized into higher-order conformational structures that allow physical interactions of regulatory elements that can be located in far distance on one chromosome or even on different chromosomes (reviewed in Cremer and Cremer 2001). Chromosome Conformation Capture (3C) and similar techniques have been developed to identify these interactions and demonstrated their impact on the regulation of transcriptional and epigenetic states (reviewed in de Wit and de Laat 2012). Hi-C, for example, allowed the investigation of the three-dimensional organization of the human and mouse genomes in embryonic stem cells and terminally differentiated cell types at unprecedented resolution (Dixon et al. 2012). Chromatin Interaction Analysis by Paired-End-Tag sequencing (ChIA-PET) is a complementary methodology that is used for the genome-wide mapping of chromatin interactions bound by specific proteins (Fullwood et al. 2009). Applying this technology to proteins generally bound by promoters or enhancers (e.g. PolII) allows the high-resolution mapping of enhancer-promoter and promoter–promoter interactions (Li et al. 2012). Data on higher-order conformational structure of a relevant cell type can thus help to unambiguously identify direct physiological target genes of functional GWAS SNPs.

### Prediction and validation of SNP-dependent differential transcription factor binding

Integrated studies often use TF-binding motifs present in DHS or DNase I footprints and overlapping ChIP-seq peaks to predict differential TF binding caused by an SNP (Schaub et al. 2012a; Maurano et al. 2012). While this is a great approach to restrict the number of SNPs associated with a certain phenotype to those that might have a causal role, like any generic approach it can also reveal a number of false positives and negatives. False hits might be the result of using databases with data generated from multiple, often for a disease or trait phenotype not relevant cell types. Many distal regulatory elements are cell type specific (Heintzman et al. 2009; Dimas et al. 2009) and thus a transcription factor that binds to a region with an SNP might not even be expressed in another cell type relevant for a certain trait or disease. Furthermore, the haplotype for the particular SNP of the cell lines used in the database is often not taken into account for these analyses. Despite huge efforts that have been taken to characterize TF-binding motifs (Badis et al. 2009; Jolma et al. 2013; Noyes et al. 2008), only for approximately half of the more than 1,000 human TFs a corresponding DNA binding motif is known, thus introducing a bias towards those. Last but not least, most motif-based approaches consider TF-binding motifs in an isolated context. However, several TFs that are part of one TF family might compete for the same motif and TFs binding in close proximity might influence each other’s affinities. For these reasons, prediction-based methods cannot yet replace the biochemical characterization of differential TF binding and activity.

Electromobility shift assays (EMSAs) are the classical method to study the interaction between a certain protein or protein domain with a particular sequence of DNA (Fried and Crothers 1981; Garner and Revzin 1981). This in vitro method, however, requires pure or highly enriched protein and, in an ideal case, an antibody that is specific for the studied TF. Chromatin immunoprecipitation (ChIP) followed by quantitative PCR of the region containing the SNP is probably the method of choice to validate the predicted differential TF binding in vivo (Stunnenberg and Vermeulen 2011). However, two major requirements need to be met. First, sufficient amounts of two disease relevant cell or tissue types that are homozygous for either variant of the SNP or alternatively a heterozygous cell type are needed. Second, a ChIP-grade antibody that recognizes the TF in question needs to be available. Furthermore, this method is a validation method that requires a priori knowledge of the binding TF. The predictions mentioned above might reveal multiple candidates each requiring a separate validation experiment. Last but not least, ChIP experiments cannot distinguish between directly and indirectly bound proteins, and without performing additional, hypothesis-driven experiments it will not reveal information about recruited co-factors and protein complexes, which would significantly contribute to our understanding of the underlying regulatory mechanism.

In the following, we will introduce recent developments in the field of mass spectrometry-based proteomics that, amongst others, will be able to overcome at least some of the obstacles mentioned above.

## Mass spectrometric characterization of functional SNPs or variants

**Fig. 1:**
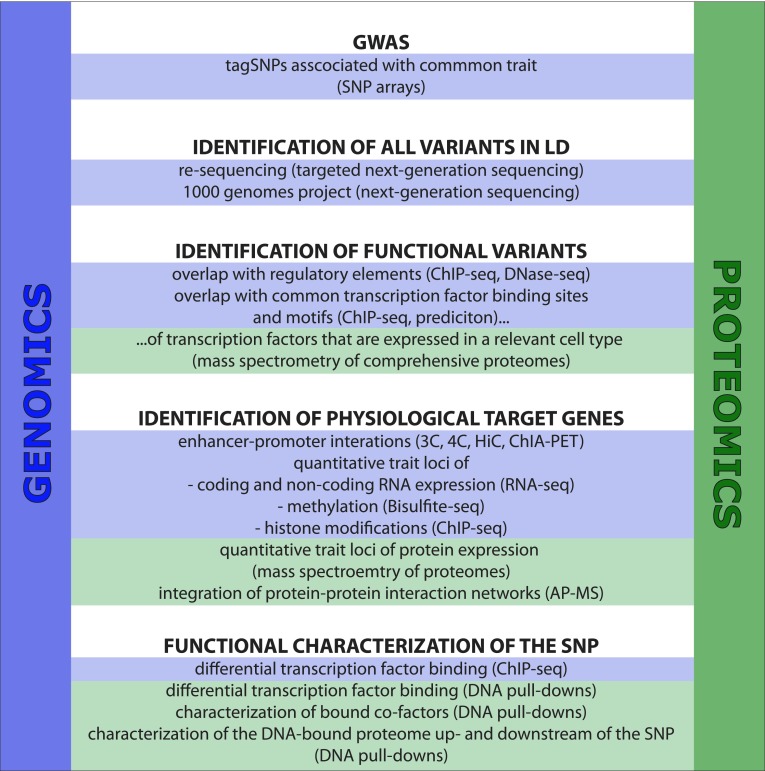
Flow-chart representing the integration of genomics and proteomics technologies for the functional characterization of common disease or phenotypic trait-associated genome variations

Due to numerous technical and computational developments during the last decade, the detection and quantification of proteins in complex mixtures by mass spectrometry have evolved to a standardized methodology (reviewed in Ahrens et al. 2010). Besides the analysis of complete proteomes (de Godoy et al. 2008) and the quantification of post-translational modifications (reviewed in Choudhary and Mann 2010), the technique has also been widely used to study protein interactions and complexes in an unbiased manner (reviewed in Vermeulen et al. 2008), thus offering an alternative to affinity purification followed by western blotting with specific antibodies. Here, we will review recent developments in the proteomics field that can be perfectly integrated in post-GWAS studies and thus contribute significantly to the identification and functional characterization of trait-associated SNPs or variants (Fig. 1).

### Integration of comprehensive protein expression data

In contrast to transcriptomics, proteomics for long had the disadvantage of not being comprehensive and requiring substantial amounts of material. The limited sequencing speed of mass spectrometers as well as the immense dynamic range of the human proteome made it difficult to identify all proteins in a reasonable time frame and with reasonable effort. Recent developments of novel methods, software and instrumentation now allow the identification of comprehensive proteomes as demonstrated for yeast a couple of years ago (de Godoy et al. 2008). In minimal amounts of human cells or tissue more than 10,000 proteins can be detected, presumably covering most of the expressed proteins (Wisniewski et al. 2013; Munoz et al. 2011). In contrast to next-generation sequencing, in which the number of reads for a certain genomic region is directly proportional to the amount of DNA or RNA present in the sample, mass spectrometry is not inherently quantitative. As a result, the detected peptide and consequently protein intensities do not represent the absolute abundance of proteins in a cell. Internal standards and computational normalization can solve this issue (Schwanhäusser et al. 2011; Picotti et al. 2009; Zeiler et al. 2012). In summary, it is now possible to obtain comprehensive proteomes with copy number information for each protein from a minimal amount of material.

As described above, approaches such as the one used in RegulomeDB integrate published epigenomic datasets from multiple cell lines to indicate which SNPs are likely to have a functional influence. In an ideal case scenario, this analysis would be done on data generated in a disease or trait relevant tissue. While large efforts are being undertaken to map epigenetic marks and DNA hypersensitivity in most human tissues (Adams et al. 2012; Chadwick 2012), ChIP-seq profiles of all TFs in all tissues are unlikely to be available in the near future. Comprehensive proteomes provide information about the presence and abundance of TFs in a certain cell type. Therefore, proteomic profiles of all tissues can serve as a filter for TF-binding predictions based on DNA motifs or ChIP-seq profiles in common cell lines. We thus strongly propose to include comprehensive and quantitative proteome mapping into large-scale epigenome mapping efforts.

### Protein quantitative trait locus analysis

It is known for more than a decade that global mRNA and protein levels do not correlate well (Gygi et al. 1999b). Reasons for this are various layers of post-translational regulation that buffer changes in transcript abundance or lead to alterations in protein abundance despite a constant transcript level. This raises the question whether polymorphisms in eQTLs have a comparable effect on transcript and protein levels.

Already in 2007, the first protein quantitative trait locus (pQTL) study was performed, analyzing the proteomes of two laboratory yeast strains and 98 segregants (Foss et al. 2007). All of these strains had also been genotyped and studied with regard to the genetic basis of variation in transcript levels (Brem et al. 2005). From this study, it became clear that loci that influence protein levels differ from those that influence transcript abundance. This emphasizes the importance of the direct analysis of the proteome. However, the proteomics technology at that time yielded limited proteome coverage. Furthermore, a very small set of proteins was quantified across all samples resulting in a strong bias towards high abundant proteins. Targeted mass spectrometry now allows the reliable quantification of a selected set of approximately 50 proteins across a broad abundance range and a large number of samples (Picotti et al. 2009). On this basis, the study of 2007 was repeated, resulting in a much more complete dataset while at the same time requiring less measurement time (Picotti et al. 2013). Novel pQTLs as well as epistatic interactions were detected. Furthermore, the authors identified two cases of co-inheritance of several independent genetic variations that influence the abundance of related proteins in a biologically coherent manner. Recently, the first human pQTL study comparing the proteome of lymphoblastoid cell lines from 95 individuals that were genotyped in the HapMap Project (www.hapmap.org) was published (Wu et al. 2013). Similar to the yeast study mentioned above, the authors stressed the limited overlap between eQTLs and pQTLs, indicating that distinct and diverse genetic mechanisms control gene expression at many different levels.

A big bottleneck in these studies, however, is the limited throughput. As the samples were measured consecutively, a still substantial amount of measurement time was required. Recent developments on the basis of isotope labels, introduced either metabolically in cell culture and/or chemically on peptide level (for detailed review, see Nikolov et al. 2012), allowed the measurement of up to 54 samples in a single experiment (Everley et al. 2013; Hebert et al. 2013). While metabolic labeling such as stable isotope labeling by amino acids in cell culture (SILAC) (Ong et al. 2002) is a very elegant, highly accurate method, it is only applicable to cells dividing in culture and thus cannot be used for the multiplexed analysis of human tissue samples. The development of multiplexing approaches such as the ones mentioned above that solely rely on chemical labeling (Boersema et al. 2009; Ross et al. 2004; Thompson et al. 2003; Gygi et al. 1999a) would therefore immensely benefit the feasibility of human pQTL studies.

### Integrating large-scale data on protein–protein interactions to interpret GWAS

**Fig. 2:**
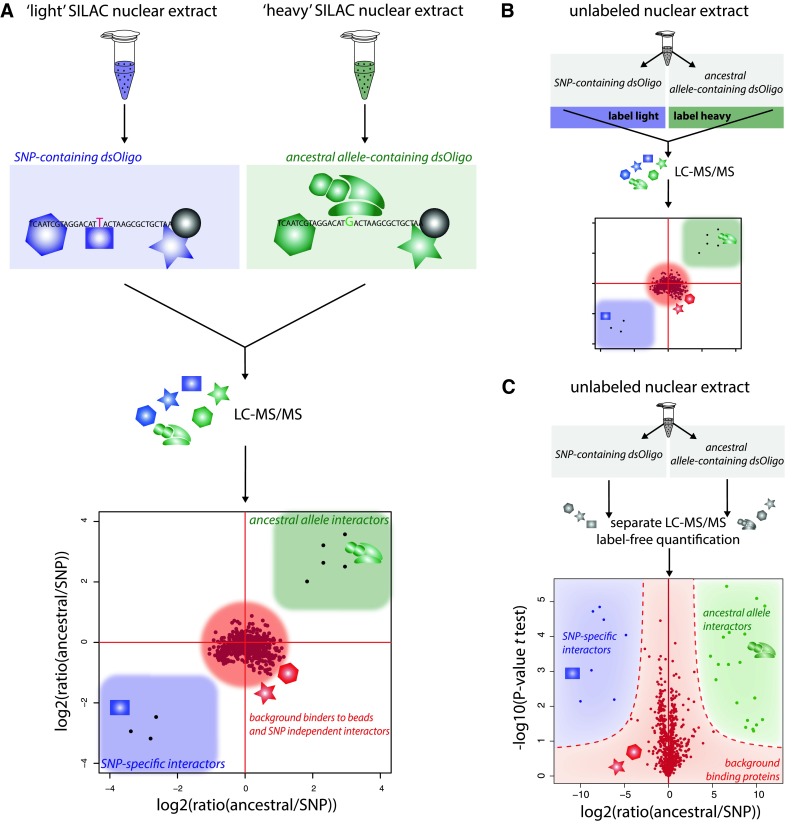
Illustration of different DNA-pulldown workflows. General workflows for the identification of SNP-dependent, dynamic DNA–protein interactions using DNA-pulldowns based on metabolic isotope labeling (**a**), chemical labeling (**b**) or label-free protein quantification (**c**). **a** A cell line of interest is cultured in medium containing light (C12N14) or heavy (C13N15) arginine and lysine. After full incorporation of amino acids into the proteome, nuclear extracts are prepared. Biotinylated oligonucleotides containing either variant of the SNP are immobilized on streptavidin beads and incubated with the light and heavy nuclear extract. Unbound proteins are removed by several wash steps. Subsequently, proteins are eluted and differentially labeled eluates are mixed prior to tryptic digestion. Peptides are separated and identified using reversed phase liquid chromatography coupled online to a mass spectrometer (LC–MS/MS). SILAC ratios from two replicate experiments are plotted against each other. Dynamic SNP interacting proteins (large or small ratio) can thus be distinguished from unspecific background binders or proteins that bind to other parts of the oligonucleotides (log2(ratio) = 0) (Mittler et al. 2009). **b** In contrast to the workflow described in **a**, a normal, unlabeled nuclear extract from cells or tissue can be used. After the pulldown, proteins are eluted and digested separately. Subsequently, peptides from both pulldowns are differentially labeled by chemically introducing isotopes at the N-termini and the arginine and lysine side chains (Ranish et al. 2003). **c** In the label-free approach, all steps, including the LC–MS/MS acquisition, are carried out separately. Peptide intensities between all runs are compared using advanced label-free quantification algorithms. Dynamic SNP interacting proteins can be identified by their differential peptide intensities and their *p* value in a *t* test based on triplicates (Hubner et al. 2010)

In recent years multiple efforts, mainly employing yeast two-hybrid screens or affinity purification followed by mass spectrometry (AP-MS) have been undertaken to construct high confidence interaction networks of human proteins (Venkatesan et al. 2009; Glatter et al. 2009; Kristensen et al. 2012; Hubner et al. 2010; Mellacheruvu et al. 2013; Sowa et al. 2009). Most AP-MS approaches are based on cell lines that express tagged versions of the proteins of interest and employ quantitative methods, such as the ones described in the following section for DNA-pulldowns, to ensure high specificity (Fig. 2). Significant efforts and resources are required to reach large-scale interaction networks including a large number of proteins. For this reason, a comprehensive interaction network including all proteins that are expressed in a certain cell of interest has still not been published.

Based on published protein–protein interaction data, there have been successful attempts to integrate these networks with GWAS or traditional linkage studies to assist the prioritization of candidates genes as well as to provide a possible functional background (Califano et al. 2012). For example, Lage and co-workers created a phenome–interactome network of protein complexes implicated in genetic disorders. Based on this, they provide numerous novel disease-causing candidate genes implicated in various diseases such as inflammatory bowel disease or Alzheimer (Lage et al. 2007). Another group developed a high confidence algorithm to in silico predict protein–protein interactions that are not yet covered by the experimental procedures mentioned above (Elefsinioti et al. 2011). Subsequently, this ‘comprehensive’ protein–protein interaction network was applied to study molecular mechanisms of neurodegenerative diseases by integrating it with relevant GWAS. This analysis provided evidence of the involvement of TOMM40 in Alzheimer’s diseases.

### DNA-pulldowns to identify and study dynamic DNA–protein interactions

Quantitative proteomics allows the study of dynamic interactions of proteins or entire protein complexes with a certain DNA sequence (Fig. 2) (Ranish et al. 2003; Mittler et al. 2009). A biotinylated, double-stranded oligonucleotide of approximately 30 base pair length containing a TF-binding motif of interest is immobilized on streptavidin beads. In parallel, the same oligonucleotide containing a point mutation in the motif is immobilized on a second set of beads serving as a control. Both DNA fragments are incubated with differentially SILAC labeled nuclear extract from a cell line of interest. In several, low stringent wash steps, unbound proteins are removed and the experiments are merged into a single tube. The DNA and the bound proteins are released from the beads by cleaving with a restriction enzyme specific for a recognition sequence included into the bait oligo sequence. Alternatively, oligonucleotides can be tagged with desthiobiotin and eluted with biotin (Butter et al. 2012). Eluates are digested and analyzed by mass spectrometry. The SILAC ratios will allow the discrimination of proteins that have a higher affinity to one of the oligonucleotides and thus to one of the SNP alleles. As mentioned above, SILAC labeling is only applicable when using extract from cells that can be grown in culture. Alternatively, chemical labeling of peptides or label-free protein quantification can be used (Hubner and Mann 2011). Recently, this method allowed the identification dynamic readers for 5-(hydroxy)methylcytosine and its oxidized derivatives, an important resource for the field of epigenomics (Spruijt et al. 2013; Bartels et al. 2011). Furthermore, a variation of this approach was developed to identify proteins that specifically interact with an RNA stem-loop of interest (Scheibe et al. 2012). In addition, complementary techniques allow the identification of proteins that are associated with a single, in vivo crosslinked and purified genomic locus (Dejardin and Kingston 2009; Byrum et al. 2012). However, due to the enormous amounts of cell material that is required for these methods it remains extremely difficult and thus is not discussed here any further.

Even though DNA-pulldowns could significantly contribute to our understanding of the molecular consequences underlying human genetic variability, to our knowledge this has so far only been shown by two studies. First, DNA-pulldowns were used to identify a repressor protein, muscle growth regulator (MGR), that specifically binds to an SNP in the intron of IGF2 and leads to enhanced muscle growth in European pigs (Butter et al. 2010). Recently, a second study that applies DNA-pulldowns to study allele-specific TF binding to SNPs that are highly associated with type 1 diabetes has been published (Butter et al. 2012). The authors could reduce the initial set of 12 associated SNPs at the *IL2RA* locus to four that showed differential binding of TFs and thus might have a functional impact on the disease. The limited interaction between the GWAS and the proteomics community might be the major reason for the minimal employment of this approach in post-GWA studies.

An intrinsic problem is the throughput of DNA-pulldowns. As mentioned above, each GWA study might reveal hundreds of rare or common SNPs that are in LD with an SNP that is linked to a certain disease. The method described above, however, is only capable of profiling 5 SNPs per day and mass spectrometer. Furthermore, at least 800 µg of nuclear extract is needed for each experiment. These experiments should ideally be performed using suitable (i.e. diseased) primary material to make sure that relevant proteins are expressed at correct levels and in the correct state (splice isoforms, post-translational modifications). However, for most primary cells and tissues it will not be possible to provide the large amount of material that would be necessary for a larger screen. We are convinced, however, that the recent development of integrated computational approaches, that limit the possible functional candidates in a set of associated SNPs, as well as efforts in downscaling and potentially multiplexing DNA-pulldowns will close the gap in near future.

While DNA-pulldowns so far have only been described to map differential binding of TFs to a specific sequence containing one or multiple nucleic acid variants, it can in theory also be used to identify the sum of TFs and co-factors that bind to a specific locus of interest. This could be achieved by quantitatively comparing the protein abundance across pulldown experiments from different genomic regions. This concept was recently used in a targeted approach to identify proteins binding to the *FLO11* promoter region (Mirzaei et al. 2013). An alternative could be a discovery-based mass spectrometric acquisition method combined with a label-free quantification algorithm. We and others have already shown this concept for protein–protein interactions (Sowa et al. 2009; Hubner et al. 2010).

## Concluding remarks

GWA studies provide important information about associations between phenotypic traits and genomic loci. Now, in the post-GWAS era, a major task is to decipher the biological processes and functional mechanisms underlying these associations. This requires the rigorous integration of large-scale, multi-dimensional data and expertise from various fields. Numerous, fruitful collaborations have already been established between researchers in genetics, genomics and epigenomics. These integrated analyses are ‘straightforward’ as they rely mainly on a similar technology platform and data output from next-generation sequencing. Other fields studying the proteome or the metabolome rely on mass spectrometric measurements and thus completely different experimental set-ups and analysis pipelines. This might be the major reason why the possibilities that proteomics research offers are so far hardly recognized and integrated in post-GWA studies.

In this review, we summarized the current ‘post-GWAS workflow’ involving the identification of variants that are in linkage disequilibrium with a GWAS variant, the identification of the functional variant among those, the identification of physiological target genes as well as the characterization of the biological mechanism underlying the functional variant. Previous studies that showed the limited correlation of mRNA and protein levels in a cell (Gygi et al. 1999b) as well as the incomplete overlap of expression and protein quantitative trait loci (Foss et al. 2007; Wu et al. 2013) stress the importance of expanding the portfolio of resources that are currently used for GWAS follow-up. Therefore, we introduced recent developments in the field of proteomics and suggested how those can be efficiently integrated in the workflow outlined in Fig. 1. For example, DNA-pulldowns followed by mass spectrometry allow the unbiased characterization of SNP-dependent protein-DNA interaction dynamics such as the altered recruitment of transcription factor complexes (Butter et al. 2012).

Clearly, the road towards fully integrative, quantitative biology to study the functional mechanisms of GWAS SNPs does not end with the integration of proteomics. The system-wide profiling of protein post-translational modifications, for example phosphorylation, glycosylation or acetylation, as well as the influence of thousands of metabolites on the phenotypic appearance of a cell provide additional, very powerful datasets that could be integrated in current post-GWAS workflows. In addition, the results obtained by the approaches described in this review will need to be followed up by extensive, more detailed functional studies involving cell and tissue models to further unravel the pathogenesis underlying a certain disease trait. Taken together, GWAS could be the basis for so far unseen collaborative efforts that provide new directions for the prevention and treatment of common diseases.

## References

[CR1] A Catalog of Published Genome-Wide Association Studies (2013). http://www.genome.gov/gwastudies. Accessed 11 May 2013

[CR2] Adams D, Altucci L, Antonarakis S, Ballesteros J, Beck S, Bird A, Bock C, Boehm B, Campo E, Caricasole A, Dahl F, Dermitzakis E, Enver T, Esteller M, Estivill X, Ferguson-Smith A, Fitzgibbon J, Flicek P, Giehl C, Graf T, Grosveld F, Guigo R, Gut I, Helin K, Jarvius J, Küppers R, Lehrach H, Lengauer T, Lernmark Å, Leslie D, Loeffler M, Macintyre E, Mai A, Martens J, Minucci S, Ouwehand W, Pelicci P, Pendeville H, Porse B, Rakyan V, Reik W, Schrappe M, Schübeler D, Seifert M, Siebert R, Simmons D, Soranzo N, Spicuglia S, Stratton M, Stunnenberg H, Tanay A, Torrents D, Valencia A, Vellenga E, Vingron M, Walter J, Willcocks S (2012). BLUEPRINT to decode the epigenetic signature written in blood. Nat Biotechnol.

[CR3] Ahrens CH, Brunner E, Qeli E, Basler K, Aebersold R (2010). Generating and navigating proteome maps using mass spectrometry. Nat Rev Mol Cell Biol.

[CR4] Altmuller J, Palmer LJ, Fischer G, Scherb H, Wjst M (2001). Genomewide scans of complex human diseases: true linkage is hard to find. Am J Hum Genet.

[CR5] Altshuler DM, Gibbs RA, Peltonen L, Dermitzakis E, Schaffner SF, Yu F, Bonnen PE, de Bakker PI, Deloukas P, Gabriel SB, Gwilliam R, Hunt S, Inouye M, Jia X, Palotie A, Parkin M, Whittaker P, Chang K, Hawes A, Lewis LR, Ren Y, Wheeler D, Muzny DM, Barnes C, Darvishi K, Hurles M, Korn JM, Kristiansson K, Lee C, McCarrol SA, Nemesh J, Keinan A, Montgomery SB, Pollack S, Price AL, Soranzo N, Gonzaga-Jauregui C, Anttila V, Brodeur W, Daly MJ, Leslie S, McVean G, Moutsianas L, Nguyen H, Zhang Q, Ghori MJ, McGinnis R, McLaren W, Takeuchi F, Grossman SR, Shlyakhter I, Hostetter EB, Sabeti PC, Adebamowo CA, Foster MW, Gordon DR, Licinio J, Manca MC, Marshall PA, Matsuda I, Ngare D, Wang VO, Reddy D, Rotimi CN, Royal CD, Sharp RR, Zeng C, Brooks LD, McEwen JE (2010). Integrating common and rare genetic variation in diverse human populations. Nature.

[CR6] Badis G, Berger MF, Philippakis AA, Talukder S, Gehrke AR, Jaeger SA, Chan ET, Metzler G, Vedenko A, Chen X, Kuznetsov H, Wang CF, Coburn D, Newburger DE, Morris Q, Hughes TR, Bulyk ML (2009). Diversity and complexity in DNA recognition by transcription factors. Science.

[CR7] Bartels SJ, Spruijt CG, Brinkman AB, Jansen PW, Vermeulen M, Stunnenberg HG (2011). A SILAC-based screen for Methyl-CpG binding proteins identifies RBP-J as a DNA methylation and sequence-specific binding protein. PLoS ONE.

[CR8] Bell J, Pai A, Pickrell J, Gaffney D, Pique-Regi R, Degner J, Gilad Y, Pritchard J (2011) DNA methylation patterns associate with genetic and gene expression variation in HapMap cell lines. Genome Biol 12(1) 10.1186/gb-2011-12-1-r10 PMC3091299 21251332

[CR9] Boersema P, Raijmakers R, Lemeer S, Mohammed S, Heck A (2009). Multiplex peptide stable isotope dimethyl labeling for quantitative proteomics. Nat Protoc.

[CR10] Botstein D, Risch N (2003). Discovering genotypes underlying human phenotypes: past successes for mendelian disease, future approaches for complex disease. Nat Genet.

[CR11] Boyle AP, Davis S, Shulha HP, Meltzer P, Margulies EH, Weng Z, Furey TS, Crawford GE (2008). High-resolution mapping and characterization of open chromatin across the genome. Cell.

[CR12] Boyle AP, Hong EL, Hariharan M, Cheng Y, Schaub MA, Kasowski M, Karczewski KJ, Park J, Hitz BC, Weng S, Cherry JM, Snyder M (2012). Annotation of functional variation in personal genomes using RegulomeDB. Genome Res.

[CR13] Brem RB, Storey JD, Whittle J, Kruglyak L (2005). Genetic interactions between polymorphisms that affect gene expression in yeast. Nature.

[CR14] Butter F, Kappei D, Buchholz F, Vermeulen M, Mann M (2010). A domesticated transposon mediates the effects of a single-nucleotide polymorphism responsible for enhanced muscle growth. EMBO Rep.

[CR15] Butter F, Davison L, Viturawong T, Scheibe M, Vermeulen M, Todd JA, Mann M (2012). Proteome-wide analysis of disease-associated SNPs that show allele-specific transcription factor binding. PLoS Genet.

[CR16] Byrum SD, Raman A, Taverna SD, Tackett AJ (2012). ChAP-MS: a method for identification of proteins and histone posttranslational modifications at a single genomic locus. Cell Rep.

[CR17] Califano A, Butte AJ, Friend S, Ideker T, Schadt E (2012). Leveraging models of cell regulation and GWAS data in integrative network-based association studies. Nat Genet.

[CR18] Chadwick LH (2012). The NIH Roadmap Epigenomics Program data resource. Epigenomics.

[CR19] Choudhary C, Mann M (2010). Decoding signalling networks by mass spectrometry-based proteomics. Nat Rev Mol Cell Biol.

[CR20] Cookson W, Liang L, Abecasis G, Moffatt M, Lathrop M (2009). Mapping complex disease traits with global gene expression. Nat Rev Genet.

[CR21] Crawford GE, Holt IE, Whittle J, Webb BD, Tai D, Davis S, Margulies EH, Chen Y, Bernat JA, Ginsburg D, Zhou D, Luo S, Vasicek TJ, Daly MJ, Wolfsberg TG, Collins FS (2006). Genome-wide mapping of DNase hypersensitive sites using massively parallel signature sequencing (MPSS). Genome Res.

[CR22] Cremer T, Cremer C (2001). Chromosome territories, nuclear architecture and gene regulation in mammalian cells. Nat Rev Genet.

[CR23] Curtis C, Shah SP, Chin SF, Turashvili G, Rueda OM, Dunning MJ, Speed D, Lynch AG, Samarajiwa S, Yuan Y, Graf S, Ha G, Haffari G, Bashashati A, Russell R, McKinney S, Langerod A, Green A, Provenzano E, Wishart G, Pinder S, Watson P, Markowetz F, Murphy L, Ellis I, Purushotham A, Borresen-Dale AL, Brenton JD, Tavare S, Caldas C, Aparicio S (2012). The genomic and transcriptomic architecture of 2,000 breast tumours reveals novel subgroups. Nature.

[CR24] Cvejic A, Haer-Wigman L, Stephens JC, Kostadima M, Smethurst PA, Frontini M, van den Akker E, Bertone P, Bielczyk-Maczynska E, Farrow S, Fehrmann RS, Gray A, de Haas M, Haver VG, Jordan G, Karjalainen J, Kerstens HH, Kiddle G, Lloyd-Jones H, Needs M, Poole J, Soussan AA, Rendon A, Rieneck K, Sambrook JG, Schepers H, Sillje HH, Sipos B, Swinkels D, Tamuri AU, Verweij N, Watkins NA, Westra HJ, Stemple D, Franke L, Soranzo N, Stunnenberg HG, Goldman N, van der Harst P, van der Schoot CE, Ouwehand WH, Albers CA (2013). SMIM1 underlies the Vel blood group and influences red blood cell traits. Nat Genet.

[CR25] de Godoy L, Olsen J, Cox J, Nielsen M, Hubner N, Fröhlich F, Walther T, Mann M (2008). Comprehensive mass-spectrometry-based proteome quantification of haploid versus diploid yeast. Nature.

[CR26] de Wit E, de Laat W (2012). A decade of 3C technologies: insights into nuclear organization. Genes Dev.

[CR27] Dejardin J, Kingston RE (2009). Purification of proteins associated with specific genomic loci. Cell.

[CR28] Dimas AS, Deutsch S, Stranger BE, Montgomery SB, Borel C, Attar-Cohen H, Ingle C, Beazley C, Gutierrez Arcelus M, Sekowska M, Gagnebin M, Nisbett J, Deloukas P, Dermitzakis ET, Antonarakis SE (2009). Common regulatory variation impacts gene expression in a cell type-dependent manner. Science.

[CR29] Dixon J, Selvaraj S, Yue F, Kim A, Li Y, Shen Y, Hu M, Liu J, Ren B (2012). Topological domains in mammalian genomes identified by analysis of chromatin interactions. Nature.

[CR30] Easton A, Webster LA, Eacott MJ (2012). The episodic nature of episodic-like memories. Learn Mem.

[CR31] Elefsinioti A, Sarac OS, Hegele A, Plake C, Hubner NC, Poser I, Sarov M, Hyman A, Mann M, Schroeder M, Stelzl U, Beyer A (2011) Large-scale de novo prediction of physical protein–protein association. Mol Cell Proteomics 10(11):M111 010629 10.1074/mcp.M111.010629 PMC3226409 21836163

[CR32] Ernst J, Kheradpour P, Mikkelsen T, Shoresh N, Ward L, Epstein C, Zhang X, Wang L, Issner R, Coyne M, Ku M, Durham T, Kellis M, Bernstein B (2011). Mapping and analysis of chromatin state dynamics in nine human cell types. Nature.

[CR33] Everley RA, Kunz RC, McAllister FE, Gygi SP (2013) Increasing throughput in targeted proteomics assays: 54-plex Quantitation in a single mass spectrometry run. Anal Chem 10.1021/ac400845e 23662842

[CR34] Foss E, Radulovic D, Shaffer S, Ruderfer D, Bedalov A, Goodlett D, Kruglyak L (2007). Genetic basis of proteome variation in yeast. Nat Genet.

[CR35] Freedman M, Monteiro A, Gayther S, Coetzee G, Risch A, Plass C, Casey G, De Biasi M, Carlson C, Duggan D, James M, Liu P, Tichelaar J, Vikis H, You M, Mills I (2011). Principles for the post-GWAS functional characterization of cancer risk loci. Nat Genet.

[CR36] Fried M, Crothers DM (1981). Equilibria and kinetics of lac repressor-operator interactions by polyacrylamide gel electrophoresis. Nucleic Acids Res.

[CR37] Fullwood MJ, Liu MH, Pan YF, Liu J, Xu H, Mohamed YB, Orlov YL, Velkov S, Ho A, Mei PH, Chew EG, Huang PY, Welboren WJ, Han Y, Ooi HS, Ariyaratne PN, Vega VB, Luo Y, Tan PY, Choy PY, Wansa KD, Zhao B, Lim KS, Leow SC, Yow JS, Joseph R, Li H, Desai KV, Thomsen JS, Lee YK, Karuturi RK, Herve T, Bourque G, Stunnenberg HG, Ruan X, Cacheux-Rataboul V, Sung WK, Liu ET, Wei CL, Cheung E, Ruan Y (2009). An oestrogen-receptor-alpha-bound human chromatin interactome. Nature.

[CR38] Garner MM, Revzin A (1981). A gel electrophoresis method for quantifying the binding of proteins to specific DNA regions: application to components of the *Escherichia coli* lactose operon regulatory system. Nucleic Acids Res.

[CR39] Genomes Project Consortium, Abecasis G, Auton A, Brooks L, DePristo M, Durbin R, Handsaker R, Kang H, Marth G, McVean G (2012) An integrated map of genetic variation from 1,092 human genomes. Nature 491(7422):56–65 10.1038/nature11632 PMC3498066 23128226

[CR40] Gibbs J, van der Brug M, Hernandez D, Traynor B, Nalls M, Lai S-L, Arepalli S, Dillman A, Rafferty I, Troncoso J, Johnson R, Zielke H, Ferrucci L, Longo D, Cookson M, Singleton A (2010) Abundant quantitative trait loci exist for DNA methylation and gene expression in human brain. PLoS Genet 6(5) 10.1371/journal.pgen.1000952 PMC2869317 20485568

[CR41] Gibson G (2011). Rare and common variants: twenty arguments. Nat Rev Genet.

[CR42] Gibson PR, Shepherd SJ (2012) Food choice as a key management strategy for functional gastrointestinal symptoms. Am J Gastroenterol 107(5):657–666; quiz 667 10.1038/ajg.2012.49 22488077

[CR43] Glatter T, Wepf A, Aebersold R, Gstaiger M (2009) An integrated workflow for charting the human interaction proteome: insights into the PP2A system. Mol Syst Biol 5 10.1038/msb.2008.75 PMC2644174 19156129

[CR44] Grossman S, Andersen K, Shlyakhter I, Tabrizi S, Winnicki S, Yen A, Park D, Griesemer D, Karlsson E, Wong S, Cabili M, Adegbola R, Bamezai R, Hill A, Vannberg F, Rinn J, Genomes P, Lander E, Schaffner S, Sabeti P (2013). Identifying recent adaptations in large-scale genomic data. Cell.

[CR45] Gygi S, Rist B, Gerber S, Turecek F, Gelb M, Aebersold R (1999). Quantitative analysis of complex protein mixtures using isotope-coded affinity tags. Nat Biotechnol.

[CR46] Gygi SP, Rochon Y, Franza BR, Aebersold R (1999). Correlation between protein and mRNA abundance in yeast. Mol Cell Biol.

[CR47] Harismendy O, Notani D, Song X, Rahim NG, Tanasa B, Heintzman N, Ren B, Fu XD, Topol EJ, Rosenfeld MG, Frazer KA (2011). 9p21 DNA variants associated with coronary artery disease impair interferon-gamma signalling response. Nature.

[CR48] Hebert AS, Merrill AE, Bailey DJ, Still AJ, Westphall MS, Strieter ER, Pagliarini DJ, Coon JJ (2013). Neutron-encoded mass signatures for multiplexed proteome quantification. Nat Methods.

[CR49] Heintzman ND, Hon GC, Hawkins RD, Kheradpour P, Stark A, Harp LF, Ye Z, Lee LK, Stuart RK, Ching CW, Ching KA, Antosiewicz-Bourget JE, Liu H, Zhang X, Green RD, Lobanenkov VV, Stewart R, Thomson JA, Crawford GE, Kellis M, Ren B (2009). Histone modifications at human enhancers reflect global cell-type-specific gene expression. Nature.

[CR50] Hesselberth J, Chen X, Zhang Z, Sabo P, Sandstrom R, Reynolds A, Thurman R, Neph S, Kuehn M, Noble W, Fields S, Stamatoyannopoulos J (2009). Global mapping of protein-DNA interactions in vivo by digital genomic footprinting. Nat Methods.

[CR51] Howie B, Fuchsberger C, Stephens M, Marchini J, Abecasis G (2012). Fast and accurate genotype imputation in genome-wide association studies through pre-phasing. Nat Genet.

[CR52] Hubner N, Mann M (2011) Extracting gene function from protein–protein interactions using Quantitative BAC InteraCtomics (QUBIC). Methods (San Diego, Calif) 53(4):453-459 10.1016/j.ymeth.2010.12.016 21184827

[CR53] Hubner N, Bird A, Cox J, Splettstoesser B, Bandilla P, Poser I, Hyman A, Mann M (2010). Quantitative proteomics combined with BAC TransgeneOmics reveals in vivo protein interactions. J Cell Biol.

[CR54] Jia L, Landan G, Pomerantz M, Jaschek R, Herman P, Reich D, Yan C, Khalid O, Kantoff P, Oh W, Manak J, Berman B, Henderson B, Frenkel B, Haiman C, Freedman M, Tanay A, Coetzee G (2009) Functional enhancers at the gene-poor 8q24 cancer-linked locus. PLoS Genet 5(8) 10.1371/journal.pgen.1000597 PMC2717370 19680443

[CR55] Johnson CP, Tang HY, Carag C, Speicher DW, Discher DE (2007). Forced unfolding of proteins within cells. Science.

[CR56] Jolma A, Yan J, Whitington T, Toivonen J, Nitta KR, Rastas P, Morgunova E, Enge M, Taipale M, Wei G, Palin K, Vaquerizas JM, Vincentelli R, Luscombe NM, Hughes TR, Lemaire P, Ukkonen E, Kivioja T, Taipale J (2013). DNA-binding specificities of human transcription factors. Cell.

[CR57] Kasowski M, Grubert F, Heffelfinger C, Hariharan M, Asabere A, Waszak SM, Habegger L, Rozowsky J, Shi M, Urban AE, Hong MY, Karczewski KJ, Huber W, Weissman SM, Gerstein MB, Korbel JO, Snyder M (2010). Variation in transcription factor binding among humans. Science.

[CR58] Klein RJ, Zeiss C, Chew EY, Tsai JY, Sackler RS, Haynes C, Henning AK, SanGiovanni JP, Mane SM, Mayne ST, Bracken MB, Ferris FL, Ott J, Barnstable C, Hoh J (2005). Complement factor H polymorphism in age-related macular degeneration. Science.

[CR59] Kristensen AR, Gsponer J, Foster LJ (2012). A high-throughput approach for measuring temporal changes in the interactome. Nat Methods.

[CR60] Lage K, Karlberg EO, Storling ZM, Olason PI, Pedersen AG, Rigina O, Hinsby AM, Tumer Z, Pociot F, Tommerup N, Moreau Y, Brunak S (2007). A human phenome–interactome network of protein complexes implicated in genetic disorders. Nat Biotechnol.

[CR61] Leinonen R, Sugawara H, Shumway M (2011) The sequence read archive. Nucleic Acids Res 39(Database issue):D19–D21 10.1093/nar/gkq1019 PMC3013647 21062823

[CR62] Li G, Ruan X, Auerbach R, Sandhu K, Zheng M, Wang P, Poh H, Goh Y, Lim J, Zhang J, Sim H, Peh S, Mulawadi F, Ong C, Orlov Y, Hong S, Zhang Z, Landt S, Raha D, Euskirchen G, Wei C-L, Ge W, Wang H, Davis C, Fisher-Aylor K, Mortazavi A, Gerstein M, Gingeras T, Wold B, Sun Y, Fullwood M, Cheung E, Liu E, Sung W-K, Snyder M, Ruan Y (2012). Extensive promoter-centered chromatin interactions provide a topological basis for transcription regulation. Cell.

[CR63] Li Q, Seo JH, Stranger B, McKenna A, Pe’er I, Laframboise T, Brown M, Tyekucheva S, Freedman ML (2013). Integrative eQTL-based analyses reveal the biology of breast cancer risk loci. Cell.

[CR64] Manolio TA, Bailey-Wilson JE, Collins FS (2006). Genes, environment and the value of prospective cohort studies. Nat Rev Genet.

[CR65] Maurano MT, Humbert R, Rynes E, Thurman RE, Haugen E, Wang H, Reynolds AP, Sandstrom R, Qu H, Brody J, Shafer A, Neri F, Lee K, Kutyavin T, Stehling-Sun S, Johnson AK, Canfield TK, Giste E, Diegel M, Bates D, Hansen RS, Neph S, Sabo PJ, Heimfeld S, Raubitschek A, Ziegler S, Cotsapas C, Sotoodehnia N, Glass I, Sunyaev SR, Kaul R, Stamatoyannopoulos JA (2012). Systematic localization of common disease-associated variation in regulatory DNA. Science.

[CR66] Mellacheruvu D, Wright Z, Couzens AL, Lambert JP, St-Denis NA, Li T, Miteva YV, Hauri S, Sardiu ME, Low TY, Halim VA, Bagshaw RD, Hubner NC, Al-Hakim A, Bouchard A, Faubert D, Fermin D, Dunham WH, Goudreault M, Lin ZY, Badillo BG, Pawson T, Durocher D, Coulombe B, Aebersold R, Superti-Furga G, Colinge J, Heck AJ, Choi H, Gstaiger M, Mohammed S, Cristea IM, Bennett KL, Washburn MP, Raught B, Ewing RM, Gingras AC, Nesvizhskii AI (2013). The CRAPome: a contaminant repository for affinity purification-mass spectrometry data. Nat Methods.

[CR67] Mirzaei H, Knijnenburg TA, Kim B, Robinson M, Picotti P, Carter GW, Li S, Dilworth DJ, Eng JK, Aitchison JD, Shmulevich I, Galitski T, Aebersold R, Ranish J (2013). Systematic measurement of transcription factor-DNA interactions by targeted mass spectrometry identifies candidate gene regulatory proteins. Proc Natl Acad Sci USA.

[CR68] Mittler G, Butter F, Mann M (2009). A SILAC-based DNA protein interaction screen that identifies candidate binding proteins to functional DNA elements. Genome Res.

[CR69] Munoz J, Low TY, Kok YJ, Chin A, Frese CK, Ding V, Choo A, Heck AJ (2011). The quantitative proteomes of human-induced pluripotent stem cells and embryonic stem cells. Mol Syst Biol.

[CR70] Nejentsev S, Walker N, Riches D, Egholm M, Todd J (2009). Rare variants of IFIH1, a gene implicated in antiviral responses, protect against type 1 diabetes. Science (New York, NY).

[CR71] Ng PC, Henikoff S (2003). SIFT: predicting amino acid changes that affect protein function. Nucleic Acids Res.

[CR72] Nikolov M, Schmidt C, Urlaub H (2012). Quantitative mass spectrometry-based proteomics: an overview. Methods Mol Biol.

[CR73] Noyes MB, Christensen RG, Wakabayashi A, Stormo GD, Brodsky MH, Wolfe SA (2008). Analysis of homeodomain specificities allows the family-wide prediction of preferred recognition sites. Cell.

[CR74] Ong S-E, Blagoev B, Kratchmarova I, Kristensen D, Steen H, Pandey A, Mann M (2002). Stable isotope labeling by amino acids in cell culture, SILAC, as a simple and accurate approach to expression proteomics. Mol Cell Proteomics.

[CR75] Paul DS, Nisbet JP, Yang TP, Meacham S, Rendon A, Hautaviita K, Tallila J, White J, Tijssen MR, Sivapalaratnam S, Basart H, Trip MD, Gottgens B, Soranzo N, Ouwehand WH, Deloukas P (2011). Maps of open chromatin guide the functional follow-up of genome-wide association signals: application to hematological traits. PLoS Genet.

[CR76] Picotti P, Bodenmiller B, Mueller LN, Domon B, Aebersold R (2009). Full dynamic range proteome analysis of *S. cerevisiae* by targeted proteomics. Cell.

[CR77] Picotti P, Clement-Ziza M, Lam H, Campbell DS, Schmidt A, Deutsch EW, Rost H, Sun Z, Rinner O, Reiter L, Shen Q, Michaelson JJ, Frei A, Alberti S, Kusebauch U, Wollscheid B, Moritz RL, Beyer A, Aebersold R (2013). A complete mass-spectrometric map of the yeast proteome applied to quantitative trait analysis. Nature.

[CR78] Ranish JA, Yi EC, Leslie DM, Purvine SO, Goodlett DR, Eng J, Aebersold R (2003). The study of macromolecular complexes by quantitative proteomics. Nat Genet.

[CR79] Raval A, Tanner SM, Byrd JC, Angerman EB, Perko JD, Chen SS, Hackanson B, Grever MR, Lucas DM, Matkovic JJ, Lin TS, Kipps TJ, Murray F, Weisenburger D, Sanger W, Lynch J, Watson P, Jansen M, Yoshinaga Y, Rosenquist R, de Jong PJ, Coggill P, Beck S, Lynch H, de la Chapelle A, Plass C (2007). Downregulation of death-associated protein kinase 1 (DAPK1) in chronic lymphocytic leukemia. Cell.

[CR80] Reddy T, Gertz J, Pauli F, Kucera K, Varley K, Newberry K, Marinov G, Mortazavi A, Williams B, Song L, Crawford G, Wold B, Willard H, Myers R (2012). Effects of sequence variation on differential allelic transcription factor occupancy and gene expression. Genome Res.

[CR81] Risch N, Merikangas K (1996). The future of genetic studies of complex human diseases. Science.

[CR82] Rivas M, Beaudoin M, Gardet A, Stevens C, Sharma Y, Zhang C, Boucher G, Ripke S, Ellinghaus D, Burtt N, Fennell T, Kirby A, Latiano A, Goyette P, Green T, Halfvarson J, Haritunians T, Korn J, Kuruvilla F, Lagacé C, Neale B, Lo K, Schumm P, Törkvist L, National Institute of Diabetes, Digestive Kidney Diseases Inflammatory Bowel Disease Genetics C, United Kingdom Inflammatory Bowel Disease Genetics Consortium, International Inflammatory Bowel Disease Genetics Consortium, Dubinsky M, Brant S, Silverberg M, Duerr R, Altshuler D, Gabriel S, Lettre G, Franke A, D’Amato M, McGovern D, Cho J, Rioux J, Xavier R, Daly M (2011) Deep resequencing of GWAS loci identifies independent rare variants associated with inflammatory bowel disease. Nat Genet 43(11):1066–1073 10.1038/ng.952 PMC3378381 21983784

[CR83] Robertson G, Hirst M, Bainbridge M, Bilenky M, Zhao Y, Zeng T, Euskirchen G, Bernier B, Varhol R, Delaney A, Thiessen N, Griffith OL, He A, Marra M, Snyder M, Jones S (2007). Genome-wide profiles of STAT1 DNA association using chromatin immunoprecipitation and massively parallel sequencing. Nat Methods.

[CR84] Ross P, Huang Y, Marchese J, Williamson B, Parker K, Hattan S, Khainovski N, Pillai S, Dey S, Daniels S, Purkayastha S, Juhasz P, Martin S, Bartlet-Jones M, He F, Jacobson A, Pappin D (2004). Multiplexed protein quantitation in Saccharomyces cerevisiae using amine-reactive isobaric tagging reagents. Mol Cell Proteomics.

[CR85] Saccone SF, Quan J, Mehta G, Bolze R, Thomas P, Deelman E, Tischfield JA, Rice JP (2011) New tools and methods for direct programmatic access to the dbSNP relational database. Nucleic Acids Res 39(Database issue):D901–D907 10.1093/nar/gkq1054 PMC3013662 21037260

[CR86] Saeed S, Logie C, Francoijs KJ, Frige G, Romanenghi M, Nielsen FG, Raats L, Shahhoseini M, Huynen M, Altucci L, Minucci S, Martens JH, Stunnenberg HG (2012). Chromatin accessibility, p300, and histone acetylation define PML-RARalpha and AML1-ETO binding sites in acute myeloid leukemia. Blood.

[CR87] Schaub MA, Boyle AP, Kundaje A, Batzoglou S, Snyder M (2012). Linking disease associations with regulatory information in the human genome. Genome Res.

[CR88] Schaub RE, Poole SJ, Garza-Sanchez F, Benbow S, Hayes CS (2012). Proteobacterial ArfA peptides are synthesized from non-stop messenger RNAs. J Biol Chem.

[CR89] Scheibe M, Butter F, Hafner M, Tuschl T, Mann M (2012). Quantitative mass spectrometry and PAR-CLIP to identify RNA-protein interactions. Nucleic Acids Res.

[CR90] Schwanhäusser B, Busse D, Li N, Dittmar G, Schuchhardt J, Wolf J, Chen W, Selbach M (2011). Global quantification of mammalian gene expression control. Nature.

[CR91] Slatkin M (2008). Linkage disequilibrium–understanding the evolutionary past and mapping the medical future. Nat Rev Genet.

[CR92] Smith LT, Lin M, Brena RM, Lang JC, Schuller DE, Otterson GA, Morrison CD, Smiraglia DJ, Plass C (2006). Epigenetic regulation of the tumor suppressor gene TCF21 on 6q23-q24 in lung and head and neck cancer. Proc Natl Acad Sci USA.

[CR93] Sowa ME, Bennett EJ, Gygi SP, Harper JW (2009). Defining the human deubiquitinating enzyme interaction landscape. Cell.

[CR94] Spruijt C, Gnerlich F, Smits A, Pfaffeneder T, Jansen P, Bauer C, Münzel M, Wagner M, Müller M, Khan F, Eberl H, Mensinga A, Brinkman A, Lephikov K, Müller U, Walter J, Boelens R, van Ingen H, Leonhardt H, Carell T, Vermeulen M (2013). Dynamic readers for 5-(hydroxy)methylcytosine and its oxidized derivatives. Cell.

[CR95] Stunnenberg HG, Vermeulen M (2011). Towards cracking the epigenetic code using a combination of high-throughput epigenomics and quantitative mass spectrometry-based proteomics. BioEssays.

[CR96] Thompson A, Schäfer J, Kuhn K, Kienle S, Schwarz J, Schmidt G, Neumann T, Johnstone R, Mohammed A, Hamon C (2003). Tandem mass tags: a novel quantification strategy for comparative analysis of complex protein mixtures by MS/MS. Anal Chem.

[CR97] Venkatesan K, Rual JF, Vazquez A, Stelzl U, Lemmens I, Hirozane-Kishikawa T, Hao T, Zenkner M, Xin X, Goh KI, Yildirim MA, Simonis N, Heinzmann K, Gebreab F, Sahalie JM, Cevik S, Simon C, de Smet AS, Dann E, Smolyar A, Vinayagam A, Yu H, Szeto D, Borick H, Dricot A, Klitgord N, Murray RR, Lin C, Lalowski M, Timm J, Rau K, Boone C, Braun P, Cusick ME, Roth FP, Hill DE, Tavernier J, Wanker EE, Barabasi AL, Vidal M (2009). An empirical framework for binary interactome mapping. Nat Methods.

[CR98] Vermeulen M, Hubner N, Mann M (2008). High confidence determination of specific protein–protein interactions using quantitative mass spectrometry. Curr Opin Biotechnol.

[CR99] Visel A, Rubin EM, Pennacchio LA (2009). Genomic views of distant-acting enhancers. Nature.

[CR100] Visscher PM (2008). Sizing up human height variation. Nat Genet.

[CR101] Wisniewski JR, Dus K, Mann M (2013). Proteomic workflow for analysis of archival formalin-fixed and paraffin-embedded clinical samples to a depth of 10 000 proteins. Proteomics Clin Appl.

[CR102] Wu L, Candille SI, Choi Y, Xie D, Jiang L, Li-Pook-Than J, Tang H, Snyder M (2013) Variation and genetic control of protein abundance in humans. Nature 10.1038/nature12223 PMC3789121 23676674

[CR103] Zeiler M, Straube WL, Lundberg E, Uhlen M, Mann M (2012) A Protein Epitope Signature Tag (PrEST) library allows SILAC-based absolute quantification and multiplexed determination of protein copy numbers in cell lines. Mol Cell Proteomics 11(3):O111 009613 10.1074/mcp.O111.009613 PMC3316735 21964433

